# A qualitative study of peer education experiences and oral pre-exposure prophylaxis use among adolescent girls and young women at high risk of HIV acquisition in Kampala, Uganda

**DOI:** 10.3389/fpubh.2025.1635462

**Published:** 2025-11-27

**Authors:** Yunia Mayanja, Ivy Kayesu, Zam Nabalwanyi, Kyriaki Kosidou, Anna Mia Ekstrom, Lazaaro Mujumbusi, Rwamahe Rutakumwa

**Affiliations:** 1 Medical Research Council/Uganda Virus Research Institute and London School of Hygiene and Tropical Medicine (MRC/UVRI and LSHTM) Uganda Research Unit, Entebbe, Uganda; 2 Department of Global Public Health, Karolinska Institutet, Stockholm, Sweden; 3 Centre for Epidemiology and Community Medicine, Region Stockholm, Stockholm, Sweden; 4 Department of Infectious Diseases/Vithusan, Södersjukhuset, Stockholm, Sweden; 5 Department of Clinical Science and Education, Södersjukhuset, Stockholm, Sweden

**Keywords:** adolescent girls and young women (AGYW), peer support/peer education, oral pre-exposure prophylaxis(PrEP), Uganda/eastern and southern Africa, IMB model, qualitative study

## Abstract

**Introduction:**

Oral pre-exposure prophylaxis (PrEP) use remains low among adolescent girls and young women (AGYW) at high HIV risk in Eastern and Southern Africa. Most peer-led interventions involve brief interaction, however peer education offering extended engagement may foster peer learning. This study explored experiences of a peer education intervention among AGYW who engaged in sex work (young FSWs) in Kampala, Uganda and examined how it influenced PrEP use.

**Methods:**

From January 2023 to February 2024, we conducted in-depth interviews (IDIs) with AGYW aged 14–24, purposively sampled from a randomized trial assessing the effect of peer education on PrEP uptake and adherence. We conducted 18 baseline IDIs to assess prior PrEP knowledge and peer education experiences, and 17 follow-ups to explore experiences of the intervention. IDIs were audio-recorded, transcribed, coded (NVivo 14) using an inductive approach. Baseline IDIs were analysed thematically and follow-ups interpreted using the situated Information-Motivation-Behavioural Skills (sIMB) model of behaviour change.

**Results:**

At baseline, AGYW had varying PrEP knowledge, no prior peer education experience and negative community perceptions hindered PrEP use. All those uninterested in PrEP at baseline did not initiate it. After the intervention, participants reported improved knowledge, motivation, and behavioural skills, though contextual barriers persisted. Peer education conveyed accurate information and dispelled myths. Motivation to use PrEP stemmed from HIV risk awareness, positive experiences and attitudes regarding PrEP use, peer influence and peer-led psychosocial support. AGYW gained behavioural skills to incorporate PrEP in daily routines, use it discreetly and maintain adherence when travelling. Non-disclosure of PrEP use was commonly used to mitigate barriers to PrEP use. Persistent contextual barriers included high mobility, concurrent use or prior negative experiences with other oral medication, stigma, partner disapproval and partner violence. Peer leader competence and confidentiality were initial concerns although no incidents were reported. AGYW preferred accessing PrEP at private, nearby facilities providing adequate health education.

**Discussion:**

Integration of peer education into PrEP programs is recommended, alongside strategies addressing contextual barriers—such as improving access in remote areas, adequate counselling when taking other oral medications, community education, partner violence prevention, and subsidies for long-acting PrEP for those unable to maintain daily use.

## Introduction

Adolescent girls and young women (AGYW) in Eastern and Southern Africa continue to have low oral pre-exposure prophylaxis (PrEP) uptake, adherence and persistence (1–4), despite continuously high HIV infection rates (5). AGYW who engage in sex work ‘hereafter referred to as young FSWs’ are at even higher risk of HIV infection compared to peers not engaged in sex work (6, 7) with HIV incidence rates (4.3–13.2%) (8–10). This has contributed to high HIV prevalence rates (average 36%) according to a systematic review and meta-analysis of studies in SSA (11). The HIV prevalence among young FSWs in Uganda ranges from 12% in a national survey of both rural and urban areas (12) to 24% in the slums of Kampala, the capital city (7). The World Health Organisation (WHO) recommends several pre-exposure prophylaxis methods for HIV prevention (13–16) of which oral PrEP remains the most widely available to AGYW in SSA. Health workers have previously provided oral PrEP services in PrEP implementation projects (1–3), but more recent projects are utilising peer-led strategies to improve PrEP outcomes among individuals at risk of HIV acquisition. For example, peer-led delivery of HIV self-test (HIVST) kits has shown to be feasible and acceptable both as a stand-alone service (17, 18) and in combination with oral PrEP (19). Furthermore, maximising peer support (information, HIVST kit-delivery, and referral) as seen among young FSWs in Kenya motivates uptake of peer-delivered HIVST kits (20). A commonality in previous studies involving peer-led strategies was that most interactions with peers were brief and mostly on individual basis. Peer support options, more specifically group peer education with sufficient interaction time for knowledge transfer and information sharing fosters peer learning and offers additional benefits for health services uptake. Peer education means sharing information and experiences among individuals with similar characteristics aimed at developing the necessary knowledge, attitudes, and behavioural skills for positive behaviour change (21, 22).

Several theories explain how peer education achieves the desired behavioural change. They include but are not limited to the *“social learning theory”* which highlights the role of peer leaders as role models who positively reinforce behaviour (23), the *“theory of planned behaviour”* which suggests that behaviour is shaped by proximal factors like a person’s attitude, subjective norms, and perceived sense of control over their actions—factors that influence behavioural intentions which in turn influence actual behaviour (24), and the “*diffusion of innovation theory”* which explains how new ideas (innovations) are propagated over time via communication channels in a social system, starting with early adopters and moving towards the broader community (25). Peer leaders may act as positive role models in education by passing on credible and acceptable information with higher success than professionals and may be more successful in educating hard to reach populations (26). Additionally, peer leaders are not seen as authority figures (27) and young people prefer peer education to discuss sensitive or insufficiently addressed topics (21). Peer education has improved sexual and reproductive health outcomes among young people in different African contexts for example, knowledge of HIV, STIs and condom use (28, 29) HIV testing and condom use (29) and contraceptive use (30). Considering young FSWs in Uganda, weekly peer education sessions combined with bi-monthly text message reminders increased uptake of quarterly HIV and syphilis testing (31). WHO recommends support groups to improve oral PrEP adherence (32) but there is limited knowledge of how this works among young FSWs. We used qualitative methods to explore experiences of peer education for oral PrEP use among AGYW at high risk of HIV acquisition in Kampala, Uganda and to understand if and how it influenced their PrEP use.

## Methods

### Study design

A qualitative study used a phenomenological approach to explore participants’ individual experiences regarding peer education, remaining open to the data and allowing themes and patterns to emerge naturally. Iin-depth interviews (IDIs) were conducted (January 2023–February 2024) within a randomised controlled trial (RCT) that assessed the effect of a peer education intervention on oral PrEP use among 394 AGYW who commonly reported sex work (young FSWs) in Kampala. The control group received health worker counselling while the intervention received health worker counselling plus monthly group peer education sessions for four consecutive months. The hypothesis of the RCT was that peer support, delivered through peer education alongside routine health worker counselling, would improve knowledge of and attitudes towards oral PrEP, thereby improving PrEP uptake and adherence. At the time of the study, daily oral PrEP was the only available PrEP modality within PrEP programmes in the country hence the RCT focus on improving oral PrEP use. Peer-led programmes that have similarly aimed to improve knowledge, motivation, and behavioural skills among FSWs (33) and MSM (34) have reported reductions in high-risk behaviour. The study followed the Consolidated Criteria for Reporting Qualitative studies (COREQ) checklist (35).

### Study setting

The study was conducted at the AIDS Information Centre (AIC) in Kampala a non-governmental organisation in Uganda that provides HIV testing and counselling (HTC), care and support to individuals and communities affected by HIV since 1990. At the AIC clinic, RCT participants, from which the sample for this qualitative study was constituted, received free health services including HTC, testing and treatment for sexually transmitted infections (STIs), male condoms, contraceptives, pregnancy testing and oral PrEP. Oral PrEP was provided under the auspices of the US President’s Emergency Plan for AIDS Relief (PEPFAR); participants who opted to get oral PrEP from other facilities after the study ended were given referrals.

### Study population, sampling and eligibility

We considered participants enrolled in the RCT for IDIs if they were assigned to the intervention group (*n* = 197). The RCT had enrolled from 20 communities in urban slums of Kampala that were known for sex work, entertainment venues and substance use. Participants assigned to the intervention group were selected because they attended the peer education intervention (unlike the control group) and therefore had rich information regarding experiences of the intervention and how these influenced their PrEP use. Since they also received the standard health worker counselling; they were better positioned to give their peer-education experiences in comparison to their experiences of the health worker counselling. They were purposively selected from the enrolment database using maximum variation sampling according to age group and their willingness to start PrEP, to capture a wide range of perspectives and experiences regarding peer education. This yielded four sampling quotas: (i) two quotas willing to use PrEP; 14–19 and 20–24 years; (ii) two quotas not willing to use PrEP; 14–19 and 20–24 years. Five IDIs were planned per quota giving 20 IDIs (10% RCT sample) but the actual number interviewed was also determined by data saturation, when additional interviewing no longer yielded new information. Selected individuals were contacted via phone and scheduled for interviews at the clinic or at another place of their convenience.

### Co-creation of the peer education intervention

The intervention was co-created with study counsellors and eight peer leaders aged 18–24 years, selected because they had been taking daily oral pills for ≥6 months, either PrEP or antiretroviral therapy (ART), hence had similar characteristics and experiences with the participants. Peer leaders were selected irrespective of health status given their experiences with PrEP and ART which share a lot in common, i.e., (drug composition, daily administration, side effects, similar packaging) hence peer leaders shared similar experiences. These peer leaders (including those living with HIV) were already involved in community HIV testing outreach activities as part of PrEP programmes and therefore already interacted with young people seeking HIV prevention services. Peer leaders were recommended by health workers from PrEP-providing facilities in central Kampala.

There were three iterative meetings to agree on intervention components and structure. In a 2-h initial brainstorming session, focus was put on areas that peer leaders and counsellors found of importance, drawing from their experience while interacting with AGYW receiving HIV prevention services. The peer leaders suggested addressing PrEP myths which were common among AGYW, sharing their personal experiences to motivate PrEP use and including role plays to enhance learning and participant-engagement. There were two follow-up meetings (1.5 h each) to refine and finalise intervention components. Two components were added as follows: peer leaders and counsellors agreed to include education since they regularly interacted with AGYW who were unaware of oral PrEP. Counsellors also recommended encouraging AGYW to share their own experiences and challenges with oral PrEP. The intervention components we included have previously been assessed in peer-led models among FSWs either as single or combined interventions and led to reduction in high-risk behaviour. These include group HIV/STI risk reduction education (33, 36) and role plays (36, 37). Furthermore, among young FSWs, sharing experiences and addressing myths improved PrEP uptake (38). Furthermore, participatory approaches with young people have previously been used to support co-design of interventions (39, 40) and improve engagement in HIV prevention services (41, 42).

**Figure 1:**
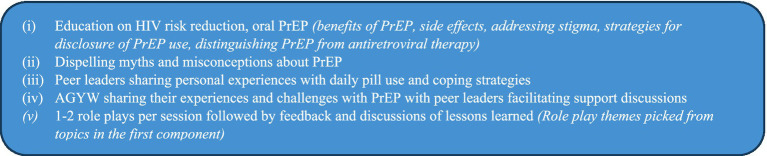
Peer education intervention components that were delivered to young FSWs by peer leaders, Kampala, Uganda (Feb–Oct 2023).

One week to intervention delivery, peer leaders were trained (2-half days) on HIV prevention for lay providers, peer leader roles and the intervention components. Peer leader roles did not involve initiating and discontinuing PrEP. PrEP could only be started, stopped and re-started in consultation with a health worker. Two groups of peer leaders (i) living with HIV, taking ART and (ii) HIV negative, taking PrEP delivered the intervention using a peer education approach. The intervention comprised five components (Figure 1).

### Data collection

A total of 35 IDIs (baseline 18; follow up 17) were conducted using a semi-structured IDI topic guide developed by the first author (YM). The guide was pilot tested using 2 interviews, refined by YM together with IK, a trained research assistant (RA). The IDIs were conducted by two RAs (IK, ZN), young females fluent in Luganda, the commonly used local language. Baseline IDIs were conducted with participants enrolled in the RCT before their participation in the peer education intervention (January–July 2023), to explore prior knowledge of oral PrEP and prior experience with peer education support for PrEP use. Interviews were conducted in a quiet private room at the AIC facility; audio recorded and took between 45 and 60 min. Data collection was discontinued by consensus after 18/20 IDIs since recurring responses and comments were noticed, during preliminary thematic data analysis, and data saturation was confirmed through team consensus. Following completion of the intervention, follow-up IDIs were conducted with the same participants (November 2023–February 2024). These aimed to explore participants’ experiences of the peer education intervention offered in the study and how it influenced their oral PrEP use. Seventeen IDs were conducted; one participant was considered as dropped off as she could not be reached by phone and never returned for her remaining RCT study visits.

### Data analysis

#### Baseline IDIs

Audio recordings were transcribed and translated to English by one of the RAs (ZN) and a social-behavioural researcher who guided the team and reviewed study documentation (LM). Transcripts were cleaned (IK, YM) through quality control checks against the audio recordings to ensure correct transcription and translation, and to correct grammatical errors. Transcripts and audio recordings were uploaded onto a secure server with controlled access. Coding was done using an inductive approach by YM, LM, IK, and ZN who also performed quality checks on transcripts that they did not code. Using open coding to generate initial codes and identify concepts, an initial coding framework was developed by all four researchers whereby each initially fully read and manually coded the same four transcripts using the inductive approach. The four transcripts were chosen by randomly selecting one transcript from each of the four sampling quotas. The four researchers independently analysed two out of the four transcripts which yielded the initial list of codes that they reviewed iteratively to ensure accuracy. The other two transcripts were then analysed to refine the codes. By way of axial coding, categories, sub-themes and themes were then developed and agreed by consensus. YM developed the coding framework in NVivo software (version 14.0) which was downloaded and checked for consistency by the team. The team then used the coding framework in NVivo to code two more transcripts to assess inter-coder reliability; further discussions for consensus were held to ensure uniformity and accuracy in the coding process. Each of the four coders was then randomly assigned 4–5 transcripts to code (*n* = 18). The entire baseline dataset was coded thematically in NVivo version 14.0. After coding, data were exported into word documents to perform line by line analysis to categorise the meaning in each line in the participants response, and a summary of each code was created.

#### Follow-up IDIs

We (YM, LM, IK, and ZN) followed the same analysis process for the 17 follow-up IDIs; axial coding identified portions of text corresponding to participants’ experiences of the peer education intervention and YM conducted the final analysis of the follow up dataset using the constructs of the sIMB model.

### Conceptual framework

**Figure 2:**
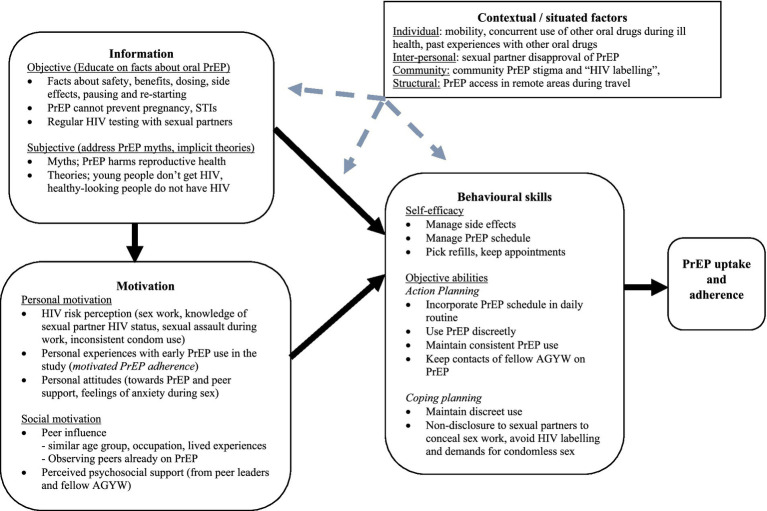
A situated information-motivation-behavioural skills model of oral PrEP uptake and adherence among AGYW who received the peer education intervention [sIMB model adapted from Dubov et al. (43)].

To understand how peer education influenced PrEP use at analysis and interpretation of findings, we applied the situated Information, Motivation and Behavioural Skills (sIMB) model for PrEP use adapted from work done among high-risk populations (43) in-turn adapted from the original IMB model by Fischer et al. (44). The model comprises of three core constructs (information, motivation, behavioural skills) that interact with each other through causal relationships to influence behaviour change (44, 45). In the PrEP context, the IMB model suggests that individuals are more likely to overcome obstacles to initiate and adhere to PrEP when they are well-informed, motivated to act on their knowledge, and possess the necessary behavioural skills to seek and initiate PrEP (43). In this qualitative study, we did not anticipate that information and motivation would have direct effects on behaviour performance as is suggested by the IMB model when behavioural skills are not complex. Given the complexity and novelty of PrEP use among study participants, behavioural skills would be key to navigate PrEP use. Amico (46) expanded the original IMB model and acknowledged that the constructs are situated within particular social, structural, and cultural contexts that influence how information, motivation, and skills are expressed and used. We therefore applied the model in context of important moderating factors external to the IMB model’s core framework (*situated factors*) influencing PrEP use in our study population. Although this study was not guided by formal theory, we used the sIMB lens at analysis to situate and contextualise the data, enabling interpretation of participants’ experiences through its core domains. In this way, we were able to make sense of findings while linking them to an existing framework hence improving sensitivity of our methods (Figure 2).

### Research team and reflexivity

We considered personal characteristics of research team members and their relationship with participants to identify pre-conceptions, ideas, experiences, values and related biases that may influence data collection, analysis and interpretation (47). The study team had diverse backgrounds (social behavioural researchers, counsellors, and medical doctor), most with extensive experience of qualitative research methods and working with young FSWs in HIV prevention studies, and all having cultural knowledge, and relevant language skill to work with the study participants. The first author’s (YM) background as a medical doctor and researcher on oral PrEP research projects among young FSWs since 2019 facilitated development of the topic guides and being a native Luganda speaker with English as her second language, helped check any inconsistencies between transcripts and audio recordings. However, YM was not involved in data collection due to her role as the principal investigator, a health care provider to study participants and age difference vis–vis the participants which could have created a power imbalance potentially biasing data collection. Authors ZN, IK conducted the IDIs. They were trained female social behavioural RAs, not health care providers, had previously worked in research projects among young FSWs and were closer in age to the participants. They were better positioned to encourage and have sincere discussions with participants hence learning from participants’ personal experiences of the peer education intervention. Additionally, the RAs were trained not to assume the counsellor role if participants became emotionally vulnerable while talking about personal experiences. They had the option to end the interview if participants became uncomfortable. Furthermore, the inductive approach used to identify themes emerging from the data enabled richer unbiased exploration of the data.

### Ethics

The study was approved by Uganda Virus Research Institute-Research Ethics Committee (GC/127/918), Uganda National Council for Science and Technology (HS2490ES), the Swedish Ethical Review Authority (Dnr 2023–05028-01), London School of Hygiene and Tropical Medicine (28193), and administrative clearance obtained from the AIDS Information Centre. Written informed consent for study participation and audio-recording of IDIs was obtained, including consent from minors identified as emancipated and / or mature minors as per the prevailing national guidelines (48). Confidentiality maintained by use of numerical codes. Trained counsellors were available if participants became emotional and could not proceed with interviews.

## Study findings

### Participant characteristics

At baseline, most participants were single, had not completed secondary education and were engaged in sex work. By follow up, nearly all had attended peer education sessions; one was lost to follow up. None of the AGYW who were unwilling to use PrEP and one who showed willingness, ever started PrEP (Table 1).

**Table 1:** Characteristics of intervention participants who were selected for baseline IDIs.

Characteristic	Categories	Numbers (n)
Age group	14–19 years	9
	20–24 years	9
Education level	Less than secondary	15
	Secondary or higher	3
Main occupation	Sex work	15
	Other	3
Marital status	Single	13
	Ever / currently married	5
Willingness to start PrEP	Yes	9
	No	9
PrEP start during the study	Yes	8
	No	10
Attended peer education	Yes	17
	No	1

Although we employed maximum variation sampling, our analysis revealed no salient differences in findings across the four sampled quotas, both at baseline and follow up.

Findings from baseline interviews are presented below with participants’ quotes to support the findings.

### Prior PrEP knowledge

Majority of AGYW stated that they had heard about PrEP before the study, however their knowledge levels varied. One noted that lack of education on PrEP use was one of the reasons hindering PrEP use among them. Some acknowledged having information gaps saying they did not understand PrEP well while others had detailed accurate information about PrEP.

*“PrEP, when you start it, for some it causes dizziness, others get diarrhoea, and some become weak. You have to swallow it in time; you swallow one pill daily. I heard people talking about it. They were saying it is good. It prevents HIV. Some people were saying that it makes people sick, that it damages some of the inner organs, but it seems like those are people who already have their illnesses. That is what I think personally”. (AGYW, 20–24 years)*

AGYW mentioned peers and other community members as their main sources of PrEP information before the study. A few mentioned first hearing about PrEP either from health workers or through educational videos when they sought reproductive health services at government health facilities.

*“My roommate was talking to her friend who seemed to have been in this job [sex work] for long. She told her to use the pills to prevent HIV, but the other one refused. She tried to convince her and told her, “If you don’t want to contract the virus, you have to use those pills. That is how I first heard about PrEP”. (AGYW, 14–19 years)*

### Prior PrEP support, experiences and preferences

All participants reported no prior experience with peer groups supporting PrEP use. They had equal preferences for PrEP support options with half preferring individualised health worker counselling and the other half peer-led support groups. A few said they would attend peer groups but would also seek further guidance from health workers as needed. Those favouring peer groups highlighted the value of sharing ideas to solve common challenges. Having similarities in age and occupation (sex work) would encourage open non-judgmental discussions hence facilitating peer learning, but they emphasised the need for well-trained peer leaders.

*“I choose the peer groups because what I am going through they [peer leaders] also go through it. You become strong when you see your friends with whom you do the same job taking those drugs [PrEP]. You can even get her contact and call her. Anything that needs counselling, you call her, and she explains it to you”. (AGYW, 20–24 years)*

Health workers were preferred because they were trusted and would keep discussions confidential. In addition, individualised sessions with health workers were seen as an opportunity for AGYW to raise all their concerns and get an expert opinion compared to the peer group sessions where several individuals would give their views. Health workers were also perceived to be better educators given their knowledge and experience of working with young people taking PrEP.

*“I would prefer to talk to the health worker because I can ask her more questions, me as an individual. A health worker is an expert and knows more than a peer leader. I can go to the health worker so that she explains to me even what the peer leader does not explain well”. (AGYW, 14–19 years)*

AGYW had varied preferences for PrEP access points including facilities near their homes, those that could be accessed privately or facilities like the study clinic where the staff knew them, PrEP was easy to access and facilities where enough time was given for health education.

*“Here [study clinic], because at least there are people I know here; instead of sending me to another place and I don’t know where to start from. It is better to get PrEP from here. There is no other place that I know”. (AGYW, 14–19 years)*

We use the sIMB model to present findings from follow-up IDIs including participants’ experiences of the peer education intervention and how it influenced PrEP use, and contextual factors that posed persistent barriers to PrEP use. Relevant excerpts from the data are included to support our findings.

### Information

#### Objective information—facts about PrEP

AGYW reported learning key facts about PrEP from peer leaders. Many who had heard about PrEP before the study had inaccurate or incomplete information; they mentioned that they benefited from peer education where several issues were clarified. Peer leaders reportedly assured AGYW that PrEP could be stopped when HIV risk decreased and restarted when risk recurred. Majority mentioned that peer leaders clarified the differences between PrEP, post exposure prophylaxis (PEP) and ART which initially confused many. Most participants acknowledged understanding information on PrEP’s safety and the temporary nature of side effects. Receiving accurate information influenced their decision to start PrEP.

*“For me, it is the signs [side effects] that was the main issue because I was scared to swallow PrEP. Many people were saying PrEP is bad; the side effects, things like that but they [peer leaders] told us, “Side effects don’t last long, with time they clear, and you will be fine” and that is what made me confident to take it [PrEP]”. (AGYW, 14–19 years)*

Of relevance to many AGYW were the peer group sessions on related health topics where peer leaders mentioned the limitations of PrEP regarding prevention of other sexually transmitted infections and unintended pregnancies. These were both common conditions among AGYW yet could not be prevented by PrEP. Majority of participants mentioned that peer leaders also advised them to continue using condoms.

*“It helped us so much to protect ourselves. Even though they [peer leaders] taught us about PrEP, they would also tell us that “even when you are swallowing those pills, you should continue using condoms to protect yourself against sexually transmitted infections”. They told us that PrEP doesn’t prevent sexually transmitted infections but only prevents HIV”. (AGYW, 14–19 years)*

#### Subjective information—myths and implicit theories

Majority of participants had received inaccurate information about PrEP in their communities and reported being sceptical about taking it when they joined the study. The most common community myth was around PrEP affecting the reproductive system which raised fears that those who took it would not be able to bear children in future. However, peer leaders dispelled this myth and AGYW gained confidence to start PrEP.

*“Back then, before I started using PrEP, I used to hear people say that ‘PrEP burns the ovaries,’ but when I attended the peer groups, they [peer leaders] taught us well. They explained to us everything, and they told us, “Those things are not true”. When they told us that, I said “let me also start taking PrEP”. (AGYW, 20–24 years)*

In addition, peer leaders also advised AGYW against implicit theories about HIV. AGYW described how peer leaders encouraged them to have regular HIV tests with their sexual partners even when their sexual partners “looked healthy.” Some AGYW who thought HIV only affected older people were reminded that young women like them were still the most vulnerable to HIV.

*“And she [peer leader] told us that not every person you see that “glitters” [looks healthy] is HIV negative. Many people you see who “glitter” are living with HIV. You should not say that someone who has HIV looks like this. You must both go and test for HIV”. (AGYW, 14–19 years)*

### Motivation

#### Personal motivation—HIV risk perception

Majority of participants reported being motivated to use PrEP due to their awareness of HIV risk, mainly because of ‘their job’ (sex work) as they commonly put it. One participant described how she had not been taking regular HIV tests thinking she had already acquired HIV, but realised she was HIV negative when tested during the study. AGYW also cited not knowing sexual partners’ HIV status, the imminent risk of sexual assault during work, and unpredictable condom use. They mentioned challenges of condom such as condom breaks, selective condom use with different types of sexual partners, and clients offering higher pay for condomless sex.

*“Firstly, I knew the problem with my job; I am at risk [HIV acquisition], the condom may burst even though it is available. Some men are forceful, like you agree on something [condom use], and he does otherwise, So I had to get those pills [PrEP] when I heard about them”. (AGYW, 20–24 years)*

#### Personal motivation—positive experiences with PrEP

AGYW stated that they were motivated to continue using PrEP due to positive personal experiences during the study. A few reported adhering well because they did not experience side effects. The majority who experienced side effects found them to be minor or significant but short-lived. Some even alluded to the earlier negative community perceptions about PrEP, affirming they were indeed untrue.

*“When I had just started swallowing, I got dizziness but there is nothing serious PrEP has done to me; I am now used to it. At first people were saying so many things about PrEP but when I started taking it, I realized that people talk about things they are not certain about. (AGYW, 14–19 years)*

#### Personal motivation—attitudes towards PrEP

Trust in PrEP effectiveness further motivated its use as participants stated that they were no longer fearful of acquiring HIV. Some explained that circumstances such as condom breaks, sexual partners refusing to use condoms and impromptu calls from some clients did not create anxiety as before because they were taking PrEP. A few reported getting more clients; one described getting more clients because she did not “bother them with condoms,” as long as she was on PrEP and contraception. Some highlighted that being on PrEP relieved them of the requirement to test with sexual partners which was unrealistic in their context of several daily sexual partners.

*“Using PrEP has helped me, I am no longer worried like before. I used to be scared and thought that maybe the condom would burst! Like I would always be anxious and wanted to go for an HIV test every time. But now I am not worried as long as I take the pills well”. (AGYW, 20–24 years)*

A few also stated their dislike for PrEP compounded by fear of side effects however trust in PrEP effectiveness enabled them to consider taking PrEP. They initially opted for condoms stating that they would only consider PrEP if their peers who took it did not get serious side effects.

*“It is not that we like it [PrEP] very much, but we have to protect ourselves. I first didn’t take it when they taught us, I said for me I am not taking it, and I asked for condoms, then I saw that others took PrEP and I said okay, ‘I will first see how they are going to be after taking it’. My friends who took it did not get any problem, then I also started”. (AGYW, 14–19 years)*

#### Social motivation—peer influence

Additional to personal motivation, peer influence through peer leaders being in the same age bracket as AGYW socially motivated them to use PrEP. They stated that this encouraged openness during discussions about sensitive topics like sexual behaviour, which some found embarrassing to discuss with health workers whom they sometimes thought of more as parents. They felt agemates understood them better. While some preferred female peer leaders for discussing sexual health, others found male peer leaders more caring and honest. A few were comfortable with any peer leader as long as they understood what they were talking about.

*“Okay, some of us were freer when talking to an age mate than the older person [health worker]. You may be shy to speak to an older person about some things [sexual behaviour]. But in the peer groups we were free because we knew these ones [peer leaders] were our age mates and they would understand us; they would not judge us”. (AGYW, 14–19 years)*

Shared lived experiences with peer leaders also motivated participants to use PrEP. Being in similar HIV risk contexts and taking PrEP enabled most of them to not only learn from others but also understand that their experiences were not different from those of their colleagues. Many said that peer leaders’ own PrEP use gave them an edge over health workers because they understood what the participants were going through unlike health workers who were not taking PrEP.

*“I benefited more from the peer groups, those ones [peer leaders] were our fellow peers, we were all taking the same pills [PrEP]. The health worker may tell you about the pills, but she doesn’t take them. But these other ones [peer leaders] experienced the same side effects that we experienced. We had the same problems and encouraged each other”. (AGYW, 20–24 years)*

Equally important were the lived experiences of peer leaders living with HIV who were also taking daily medication (ART) as their experiences were cautionary tales and reminders for AGYW to take PrEP seriously.

*“I thought about the way they were using their stories as examples. They were peer leaders living with HIV, they told us that, “you should take PrEP, if you don’t take PrEP you are going to be like me who got HIV”. When I heard that I had to take it [PrEP] every day”. (AGYW, 20–24 years)*

Peer influence as a social motivator for PrEP use also manifested through observation of peers already taking PrEP. Participants reported that this motivated them because their peers were healthy and continued to take PrEP without health problems, contrary to prior negative community perceptions that had initially hindered PrEP use. One participant also mentioned being encouraged by a peer leader who had a child while being on PrEP. Peer leaders had been taking PrEP for a longer period, they were seen as role models and enabled many, including those who were initially sceptical about PrEP, to gain confidence in using PrEP.

*“At first, I didn’t take it [PrEP] because people said bad things about it. But while teaching us here, they [peer leaders] showed me that it is okay, and those who were taking it I was seeing them; those who were teaching us [peer leaders]. So, I had to be strong and start because I could see that the condition they were in [physical health] was not what I had been hearing outside [in the community]”. (AGYW, 14–19 years)*

#### Social motivation—perceived psychosocial support

Perceived psycho-social support from peer leaders motivated PrEP use among participants. Many described receiving emotional and social support in a friendly, non-discriminatory environment where everyone contributed to discussions. Peer leaders were seen as welcoming, calm, and patient, often continuing discussions beyond sessions. Some participants appreciated follow-up calls to check on pill adherence and described peer leaders as caring. After the intervention, more participants preferred peer support compared to baseline.

*“Firstly they [peer leaders] were always free with us. Secondly, they were doing everything with care. We could disturb them any time asking questions and they always responded in a gentle way. They would call and ask; ‘did you take your pills?’ ‘Did you do this?’; they cared that we took the pills”. (AGYW, 20–24 years)*

Participants reported being motivated by psychosocial support from fellow AGYW; they reminded each other to take PrEP and pick up refills. They mentioned exchanging phone contacts for mutual support. They further pointed out that sharing challenges in group sessions led to AGYW offering practical advice to their peers using their own experiences. Some also shared accounts of fellow AGYW encouraging them to attend group sessions.

*“Even today I was not going to come, I was late, but my friend called, and she told me “I have not seen you here. They have told me that your phone was off”. We used to take our friends’ contacts; so that when one comes and doesn’t see me, she calls me. The group they put me in helped me a lot”. (AGYW, 14–19 years)*

Regardless, a few AGYW were concerned about sharing information in groups because in their opinion, the groups were not as confidential as the individualised health worker counselling. In effect, some AGYW did not derive motivation from the peer groups and preferred to be supported by health workers.

*“I could tell the health worker everything, something which I was afraid to do the other side [peer groups]. Like I told you, the girls can talk and expose you. So, I could not be so open. I could not say what was on my heart among the girls”. (AGYW, 20–24 years)*

### Behavioural skills

#### Self-efficacy

Some AGYW demonstrated self-efficacy, feeling confident in managing daily PrEP use and therefore made personal decisions to start PrEP. Conversely, those with low self-efficacy did not start PrEP, citing concerns about side effects, daily dosing, and lifestyle challenges like busy or unpredictable work schedules that would disrupt adherence and picking PrEP refills.

*“No, I have not [started PrEP] but I hope to start. I had things to do, I was up and down, and I couldn’t, and they say it’s not good to miss doses. So, you must be sure that when you start taking them you are going to swallow them. I have been busy and wasn’t going to manage PrEP”. (AGYW, 20–24 years)*

#### Objective abilities

Participants also demonstrated behavioural skills through objective abilities like *action planning* and *coping planning* for PrEP use as discussed below.

They reported receiving practical advice on managing PrEP schedules, disclosure, stigma, and side effects. Through action planning, majority linked PrEP use to daily routines to support adherence. Those lacking behavioural skills, such as incorporating PrEP into their routines, discreet use and maintaining use over time, avoided starting due to fear of missing doses or not taking pills at all. Many aligned PrEP with mealtimes or bedtime as reminders and keeping peer phone contacts helped reinforce adherence. Several participants reported moving out of the study area but some were not affected because they continued PrEP through alternative providers.

*“I was taking the pills well. There are some months when I didn’t come here, but I didn’t miss PrEP. It got finished when I was in the village, but I was able to find health workers who were giving out PrEP, I didn’t want to miss, and I got some from there which I took until I came back here”. (AGYW, 14–19 years)*

Many participants described developing coping strategies to manage barriers to PrEP use (coping planning). Some maintained discreet use when staying with regular partners, avoiding disclosure due to fear of being labelled as having HIV, revealing their engagement in sex work or experiencing intimate partner violence (IPV). However, one participant described getting into a situation of unintentional PrEP use disclosure that led to IPV from a regular sexual partner. Non-disclosure was also a deterrent to unprotected sex from partners if they became aware of AGYWs’ PrEP use. Similarly, one participant explained how discreet PrEP use was driven by sexual partners’ demands for condomless sex and disregarding HIV testing; AGYW therefore prioritized their own personal protection against HIV.

*“No one knows about it because they are not the ones who told me to take it [PrEP]. I take for personal protection because when most of them get to know that you take PrEP, they want you to have unprotected sex with them. So, if you don’t tell it to anyone, it is to your advantage”. (AGYW, 14–19 years)*

*“I cannot [disclose PrEP use to sexual partners] because they are less concerned. It is my life and it’s me to make sure that I remain fine. They [sexual partners] don’t care, they say that ‘those ones are on the job [sex work], it’s up to them to protect their lives’. So, when he comes and tells me that “I want to have sex with you without a condom” and doesn’t know my status [HIV status] that means he has his problems. They are not concerned about whether you have HIV or not. It is up to me to be careful. (AGYW, 20–24 years)*

*“The day he saw my pills [PrEP], he confronted me and asked me ‘Do you have HIV?’ Then he got mad. On that day, it was time for me to take my PrEP, I got them to swallow and that is how he saw them. He quarreled and abused me”. (AGYW, 14–19 years)*

### Contextual/situated factors

Despite receiving accurate information, being motivated and having behavioural skills, some participants faced barriers to PrEP use at multiple levels. Individual challenges included frequent mobility, concurrent use of oral medication for other illness or conditions and past negative experiences with oral drugs which they said made them ‘feel bad’. Others paused PrEP when they moved to areas without access while those prescribed other oral medications for illnesses prioritized treatment over PrEP due to pill burden. Interpersonal barriers included disapproval from sexual partners. Community-level stigma labelled PrEP users as living with HIV, deterring use. These obstacles hindered PrEP use among those who desired to take it.

*“I have been using it [PrEP] but now I have decided to pause it because I am taking too many drugs. The last time I was here, I was given drugs for an infection. When I went to the hospital for a scan they found out that I also had [condition X] and they gave me drugs because I did not want to get injections. When I thought of adding PrEP, I saw that the drugs would be too much for my body to handle”. (AGYW, 20–24 years)*

*“PrEP is good, and I can use it but what I fear most is someone seeing me swallowing it and thinking that I have HIV. And they can say that “you were showing off, now where did you get HIV from”. But now, I think I need it [PrEP], I don’t know. I think I just need to talk to someone (AGYW, 14–19 years)*

*“I got a trip; I had work in District X [a remote part of the country]. I travelled with the pills though I didn’t realize that they would not be enough. I was unable to estimate the period we were going to spend there. So, the pills got finished; I was very far, and I had no access to PrEP so I decided to pause. When I came back I re stared PrEP”. (AGYW, 20–24 years)*

## Discussion

This qualitative study done among AGYW who mainly identified as sex workers explored prior PrEP knowledge and experiences with peer education for oral PrEP use. At baseline, AGYW had varying levels of knowledge about PrEP, but none had prior exposure to peer education for oral PrEP use. We found that peer education enhanced PrEP-related knowledge, motivation, and behavioural skills; however, contextual barriers continued to impede PrEP use for some participants and none of those who were unwilling to use PrEP at baseline started PrEP. Concerns about confidentiality within peer groups and the competence of peer leaders were common during baseline interviews however, there were no reports of actual breach of confidentiality during follow up interviews. Besides, participants got used to each other and to the peer leaders, interacting freely due to shared lived experiences. However, those with reservations about confidentiality opted to consult health workers rather than share within the peer groups. Participants expressed a preference for nearby PrEP facilities offering privacy and adequate health education. No differences in experiences were observed across the purposively sampled quotas, likely due to shared contextual factors given that AGYW were recruited from urban slums in Kampala with similar characteristics and attended the same intervention during the study. Therefore, despite varied background characteristics they shared common experiences.

According to the IMB model, information that is directly relevant to the performance of health behaviour and that can be easily enacted by an individual in their social ecology is a critical determinant of behaviour performance (44). We found that most AGYW had heard about PrEP but had varied knowledge levels. Inaccurate information about PrEP and myths were also common and hindered PrEP use as previously reported among young FSWs (49). Additional studies that included young FSWs in SSA corroborate our findings. According to the authors, having PrEP knowledge or information (50, 51) and positive attitudes towards PrEP (52) improve acceptability and use of oral PrEP. Similarly, studies in Zambia and Uganda that have included different high-risk populations, i.e., FSWs, MSM, injecting drug users (IDUs), fisherfolk, echo the same findings with reports of PrEP misinformation being common, and negatively impacting PrEP uptake (53, 54). On the contrary, one Tanzania study showed low PrEP use among FSWs despite improved knowledge and consistently high willingness to use PrEP during 12-month follow up (55) suggesting the need for additional strategies to translate willingness into PrEP use. Indeed, strategies like peer support have improved PrEP use and persistence among FSWs in Kenya and Uganda (56, 57) and in Nigeria, high risk populations including FSWs proposed trained peer educators and HIV-test counsellors to provide information and referrals to PrEP providing facilities with peer-led PrEP provision (58). In our study, after receiving accurate information about PrEP during peer groups, participants’ confidence to start PrEP was enhanced. Increased communication and interactions among peers have fostered support systems for PrEP use among AGYW at high HIV risk in Southern Africa (59) and improved PrEP knowledge and uptake among black MSM elsewhere (60), suggesting that frequent interaction during peer support activities improves knowledge of PrEP and may improve PrEP uptake and adherence.

Similar to our findings, community settings have been identified as the largest source of inaccurate information about PrEP (61) leading to community-level PrEP stigma and low psychosocial support for PrEP use among FSWs aged ≥18 years (62) and other high risk AGYW who do not identify as sex workers (63, 64). Indeed, we found that negative community perceptions perpetuated inaccurate information that hindered PrEP use before joining the study. Considering that some FSWs prefer community-based PrEP services (52), and that these have demonstrated high retention of FSWs on PrEP (65), these findings suggest that PrEP education should not only target end-users but also their communities, to address community-level barriers. Community engagement and stakeholder involvement were identified facilitators of PrEP uptake among high-risk AGYW in Kenya (66). Community and stakeholder involvement allows for implementation of differentiated service delivery (DSD) models for PrEP which can further be tailored to population sub-groups including at-risk AGYW who do not identify as sex workers, and other high-risk populations. These models de-medicalize, simplify and decentralise PrEP services, making them more accessible to individuals with various service delivery preferences. These include but are not limited to integration into other existing health programmes, peer-led, pharmacy based and mobile health approaches which are acceptable for oral PrEP (67–69). Facility based models that incorporate mobile health are preferred by a section of FSWs in Uganda (65).

Motivation is an additional critical determinant of behaviour performance among well informed individuals (70). We found that participants were personally motivated by high HIV risk perception, their initial PrEP experiences during the study and attitudes towards PrEP. Positive attitudes have been associated with PrEP use (52) and uptake of other HIV prevention services (71) in studies that included young FSWs while HIV risk perception was positively associated with PrEP uptake among AGYW in South Africa with several behavioural risk including ever engaging in transactional sex (72). In the current study, young FSWs identified contexts such as multiple sexual partnerships, inconsistent condom use and being unaware of sexual partners’ HIV status that influenced them to start and continue PrEP use. The minor and/ or temporary side effects they got when they started PrEP further motivated PrEP persistence. Contrary to previous literature showing side effects as factors for PrEP discontinuation among young FSWs (3), shared experiences and practical advice from peer leaders in our study influenced many participants to cope with the temporary side effects. As evidenced in this and other studies that found positive associations between peer support and PrEP uptake (65) and continuation (56) among FSWs, and PrEP disclosure among AGYW engaged in transactional sex (73). Peer-led strategies should be integrated into FSW PrEP programmes to support PrEP use. Considering this group of young FSWs, trust in PrEP effectiveness reduced anxiety during sex further motivating continued PrEP use; they were less worried about condom breaks and the impracticality of taking HIV tests with every sexual partner before sex. Social motivation for PrEP use was mainly through peer influence and psycho-social support. Participants did not observe ill health either among peer leaders who had taken PrEP for longer periods or fellow AGYW who started PrEP during the study. Being positively influenced by peers in similar circumstances is similarly reported in a study that assessed peer-delivery of HIV self-test kits and PrEP in another Ugandan study among young FSWs (19). We found that peer leaders created a friendly non-discriminatory environment and were responsive to AGYWs’ concerns, and that fellow AGYW reminded their peers to take PrEP and attend the peer groups. Indeed, after the intervention, AGYW reported having higher preference for peer-led group support. PrEP support through peer mentors similarly facilitated PrEP use among high-risk AGYW enrolled in the DREAMS initiative in Kenya (66). Notably, peer leaders living with HIV cautioned AGYW about the outcomes of casual PrEP use. PrEP support and delivery systems which utilise peer leaders are facilitators of PrEP use (66) therefore peer leaders should be integrated into PrEP programmes to support young FSWs who struggle with PrEP.

The behavioural skills construct of the IMB model explains the ease or difficulty with which well-informed and well-motivated individuals engage in health promotion behaviour. We found that nearly all AGYW attended peer support sessions but only those with self-efficacy used PrEP. Occupation-related factors like frequent abrupt movements, and busy unpredictable schedules common with the sex work trade were barriers to PrEP use self-efficacy in our study. Likewise, high mobility and unpredictable schedules of FSWs hindered adherence among FSWs on PrEP (56) and ART (74). In the broader context of HIV where prevention of mother-to-child-transmission of HIV is also an important prevention strategy among women, a positive association between high self-efficacy and PrEP use was found among pregnant and post-partum women in Eswatini (75). While AGYW showed behavioural skills to start and maintain PrEP use through discreet use, incorporation into daily routines and finding alternative providers when they travelled, they also had skills to overcome barriers to PrEP use. AGYW had to navigate stigma and disclosure. PrEP disclosure enables reminders for PrEP and improves adherence among AGYW (76, 77) however, we found that non-disclosure of PrEP use was common among AGYW mainly to mitigate the associated negative consequences including: social stigma and “HIV labelling” (49, 63, 73, 75, 77), conflict with or violence from sexual partners (73, 78–80) and being labelled as having multiple sexual partners (49, 63, 75, 77). Given the context of AGYW we enrolled who were involved in sex work, peer-led support groups provide an appropriate platform for disclosure and peer learning, however confidentiality concerns should be addressed.

Contextual (situated) factors negatively influenced PrEP use despite participants having information, motivation and the required behavioural skills. High mobility has been associated with low PrEP adherence and persistence among FSWs (56) and high-risk AGYW who start PrEP (59) and persistence; we found that lack of PrEP access in some of the areas where AGYW move to hindered PrEP use. Hence PrEP services should be more accessible in remote regions particularly at known sex work hot spots to enable mobile individuals maintain PrEP use. In fact, long-acting PrEP formulations may be more appropriate solutions for the contextual factors that we found. Long-acting PrEP maintains discreet use for young FSWs who are mobile, fail to incorporate PrEP in their lifestyle, cannot cope with oral drugs and those with unsupportive regular sexual partners. Besides, young FSWs and other high-risk AGYW have shown higher preference for long-acting PrEP methods (81–83). Injectable PrEP formulations have shown efficacy (84, 85), and long-acting oral PrEP if found safe and efficacious in clinical trials will lessen the burden for those who prefer oral formulations. Nonetheless, long-acting PrEP will likely realise more HIV prevention benefits if incorporated within DSD models and tailored to population sub-groups. More effort to sensitise male sexual partners may also facilitate PrEP use. However, this has only been tested within the DREAMS project that included intimate partner violence prevention and gender norms education for male sexual partners (66) hence applies to sexual partners who have prior sensitization. Integration of PrEP with other sexual and reproductive health services is also highly relevant for AGYW. PrEP use should be considered and mentioned during counselling and when prescribing oral drugs for contraception, STIs or other common illnesses.

### Strengths and limitations

Although we did not collect participants’ feedback on the study findings, we took steps to avoid mis-representing their views by audio-recording the interviews, transcribing them verbatim, and independently verifying the transcripts against the recordings. In addition, we used quotes to support our findings with participants’ own words hence improving credibility of findings. The IMB model assumes similar information, motivation, and behavioural skills across individuals, which does not always hold true as was the case in our study and would require tailored information. Nevertheless, the peer education components were inclusive, reaching those with no prior knowledge and, importantly, improved the understanding of the majority who initially held inaccurate information. Similar findings across sampling quotas strengthens the credibility and transferability of our findings since they are relevant to diverse groups, and we included a reflexivity statement showing how we mitigated potential biases in the study. This study is among the few that have examined how peer education influences PrEP use among young FSWs. By incorporating contextual factors, such as mobility and PrEP access during travel, concurrent use of other oral medication, regular partner support and community stigma, our study also provides insights into potential barriers external to the IMB model that must be considered to enhance PrEP use, hence HIV prevention among AGYW at increased risk of HIV acquisition.

## Conclusion

Peer-led group education improves knowledge, motivation and behavioural skills for PrEP use among young FSWs, but contextual factors which pose additional berries must be addressed. FSW-PrEP programmes should strengthen group-based peer education activities and prioritize training and integrating more peer leaders into the health workforce to support their peers. Health workers should also provide appropriate counselling to those who are prescribed other oral medications while using PrEP to prevent non-adherence. Given the negative impact of community perceptions on PrEP use, community-wide education efforts including IPV prevention are essential to remove social and cultural barriers. PrEP services should be made more accessible, particularly in remote areas, to prevent PrEP use interruption among highly mobile individuals. Additionally, expanding access to long-acting PrEP options should be prioritized, as these offer a suitable alternative for young FSWs who may not prefer daily oral PrEP and help address the contextual challenges that hinder discreet and consistent use, even among those motivated to adhere to daily oral PrEP.

## Data Availability

The raw data supporting the conclusions of this article will be made available by the authors, without undue reservation.
